# Localization of proteins in the cell wall of *Mycobacterium avium *subsp. *paratuberculosis *K10 by proteomic analysis

**DOI:** 10.1186/1477-5956-8-21

**Published:** 2010-04-08

**Authors:** Zhiguo He, Jeroen De Buck

**Affiliations:** 1 Department of Production Animal Health, Faculty of Veterinary Medicine, University of Calgary, 3330 Hospital Drive NW, Calgary, AB T2N 4N1, Canada

## Abstract

*Mycobacterium avium *subsp. *paratuberculosis *is a pathogen which causes a debilitating chronic enteritis in ruminants. Unfortunately, the mechanisms that control *M. avium *subsp. *paratuberculosis *persistence during infection are poorly understood and the key steps for developing Johne's disease remain elusive. A proteomic analysis approach, based on one dimensional polyacrylamide gel electrophoresis (SDS-PAGE) followed by LC-MS/MS, was used to identify and characterize the cell wall associated proteins of *M. avium *subsp. *paratuberculosis *K10 and an cell surface enzymatic shaving method was used to determine the surface-exposed proteins. 309 different proteins were identified, which included 101 proteins previously annotated as hypothetical or conserved hypothetical. 38 proteins were identified as surface-exposed by trypsin treatment. To categorize and analyze these proteomic data on the proteins identified within cell wall of *M. avium *subsp. *paratuberculosis *K10, a rational bioinformatic approach was followed. The analyses of the 309 cell wall proteins provided theoretical molecular mass and p*I *distributions and determined that 18 proteins are shared with the cell surface-exposed proteome. In short, a comprehensive profile of the *M. avium *subsp. *paratuberculosis *K10 cell wall subproteome was created. The resulting proteomic profile might become the foundation for the design of new preventive, diagnostic and therapeutic strategies against mycobacterial diseases in general and *M. avium *subsp. *paratuberculosis *in particular.

## Introduction

*Mycobacterium avium *subsp. *paratuberculosis *is a member of the *M. avium *complex, next to three other subspecies *M. avium *subsp. *hominissuis, Mycobacterium avium *subsp. *avium *and *M. avium *subsp. *silvaticum *and the species *M. intracellulare*. *M. avium *subspecies *hominissuis *and *M. intracellulare *are widely distributed in the environment and also inhabit healthy animal and human intestines, but do not usually cause disease unless the host is debilitated or immunocompromised. *M. avium *subsp. *paratuberculosis*, in contrast, is a pathogen which causes a debilitating chronic enteritis in ruminants[1] and has been implicated in Crohn's disease in humans [2]. Unfortunately, the mechanisms of virulence that control *M. avium *subsp. *paratuberculosis *persistence during infection are poorly understood and the key steps for developing paratuberculosis remain elusive. The current challenge is to identify elements that are essential for virulence and survival of the bacterium during infection, especially those that influence the immune responses against *M. avium *subsp. *paratuberculosis*.

A characteristic feature of mycobacteria is the thick, waxy cell wall, a highly impermeable outer surface, which enables mycobacteria to survive in extreme environmental conditions and the presence of antibiotics. This cell wall contains 60% lipid, which confers on it the properties of acid fastness (the ability to resist decolorization by acidified alcohol), hydrophobicity, and increased resistance to chemicals (e.g. chlorine) and physical processes (e.g. pasteurization)[3].

Bacterial surface proteins play a fundamental role in the interaction between the bacterial cell and its environment [4-6]. They are involved in adhesion to and invasion of host cells, in sensing the chemical and physical conditions of the external milieu and sending appropriate signals to the cytoplasmic compartment, in mounting defenses against host responses and in toxicity. In this study, we also aimed to identify surface-exposed proteins of *M. avium *subsp. *paratuberculosis *K10 using a proteolytic digest of the bacterial surface followed by mass spectrometry. In previous studies, this enzymatic 'shaving' technique resulted in the identification of many surface exposed proteins [7-9].

The goal of this study was to comprehensively identify all cell wall associated and cell surface exposed proteins of *M. avium *subsp. *paratuberculosis *K10 to support vaccine development and pathogenesis studies.

## Materials and methods

### Bacterial strain and growth conditions

*M. avium *subsp. *paratuberculosis *K10 was grown in Middlebrook 7H9 broth (Becton Dickinson, Oakville, ON, Canada) supplemented with 0.5% glycerol, 0.05% Tween 80, 2 μg/ml of mycobactin J (Allied Monitor, Fayette, MO, US), and 10% oleic acid albumin dextrose complex (OADC, Becton Dickinson) until mid-exponential growth phase. The culture was harvested by centrifugation for 10 min at 10 000 × g at 4°C and washed three times with ice-cold phosphate buffered saline (PBS) (pH7.4). The pelleted cells were frozen at -80°C until needed.

### Cell wall proteins preparation

The extraction of cell wall proteins from *M. avium *subsp. *paratuberculosis *K10 was carried out according to Mandana *et al*. with minor modification [10]. Cells were harvested at 4400 × g and washed with NaCl solution (0.16 M). The weight of wet cells was determined and for each gram of bacteria one ml lysis buffer (0.05 M potassium phosphate, 0.022% (v/v) β-mercaptoethanol, pH 6.5) was added. Lysozyme (Roche, Mississauga, ON, Canada) was added to the cells to a final concentration of 2.4 mg/ml. The cells were then incubated at 37°C for 2 h. Subsequently, cells (maintained in screw cap Eppendorf tubes) were disrupted with a bead beater (Biospec products, USA) for 4-6 times (1.5 min each time, ice cool down at intervals). The lysates were subjected to a low speed centrifugation at 600 × g to remove unbroken cells. Centrifugation was repeated 3 to 5 times for 40 min at 22,000 × g to pellet the cell walls. All pellets were resuspended and pooled. A second cell lysis, equal to the first, was performed on the pooled pellet. A single centrifugation at 22,000 × g gave the pellet of cell wall fraction. The pellet was resuspended in PBS buffer and centrifugated at 22,000 × g, then stored frozen at -80°C.

### Bacterial surface digestion

Procedure was carried out according to Guido Grandi *et al*[7] with some modifications. Bacteria were harvested from culture at an OD_600 _of 0.4 (exponential phase) by centrifugation at 3,500 × g for 10 min at 4°C, and washed three times with PBS. Cells were resuspended in one-hundredth volume of PBS containing 40% sucrose (pH 7.4). Digestions were carried out with 20 mg proteomic grade trypsin (Sigma-Aldrich, Oakville, ON, Canada) in the presence of 5 mM DTT, for 30 min at 37°C. A control experiment in parallel was carried out. Briefly, we incubated *M. avium *subsp. *paratuberculosis *K10 cells in the "trypsin shaving" incubation buffer without trypsin for 2 hours. The digestion mixtures were centrifuged at 3,500 × g for 10 min at 4°C, and the supernatants (containing the peptides) were incubated at 37°C for around 12~14 hrs for full digestion after being filtered using 0.22 μm pore-size filters (Millipore, Etobicoke, ON, Canada). Protease reactions were stopped with formic acid at 0.1% final concentration. Peptide fractions were concentrated with a Speed-vac centrifuge (Savant), and kept at -20°C until further analysis.

### Sample digestion

Protein sample was separated by 12.5% sodium dodecyl sulfate polyacrylamide gel (SDS-PAGE), run for 1 h at 30 W, then for 4.5 h at 180 W. The gels were coomassie stained and the lane corresponding to the cell wall proteins was cut into 6 equal pieces. The gel pieces were individually in-gel digested as described previously with some modifications [11]. Briefly, after in-gel digestion using trypsin, the digested solution was transferred into a clean 0.6 ml tube. Fifty microliters of 50% acetonitrile (ACN)/5% formic acid (FA) was added to the gel pieces and sonicated for 30 min. This extraction procedure was repeated three times, and a total of 150 μl of extracts was collected. All extracts were pooled and concentrated to less than 10 μl using an SPD 2010 SpeedVac system (Thermo Electron, Waltham, MA). Thereafter, the sample was diluted with 0.1% FA in HPLC water to 100 μL for direct LC-MS/MS analysis or reconstituted with trifluoroacetic acid (TFA) to a final concentration of 0.1% and subjected to sample cleanup steps using C18 ZipTips (Millipore) prior to LC-MS/MS analysis. The C18 ZipTips were conditioned with 100% ACN and then equilibrated three times with 0.1% TFA. The peptides were bound to the ZipTip pipet tip by aspirating and dispensing the sample for at least 15 cycles, washed with 0.1% TFA, and eluted by 20 μL of elution buffer (75% ACN, 0.1% TFA).

### Protein identification by LC-MS/MS

Digests were analyzed using an integrated Agilent 1100 LC-ion-Trap-XCT-Ultra system fitted with an Agilent ChipCube source sprayer. Injected samples were first trapped and desalted on a Zorbax 300 SB-C18 Precolumn (5 μm, 5 × 300-μm inside diameter; Agilent) for 5 min with 0.2% formic acid delivered by the auxiliary pump at 0.3 μl/min. The peptides were then reverse eluted from the trapping column and separated on an analytical Zorbax 15 cm-long 300SB-C18 HPLC-Chip 0.3 μl/min. Peptides were eluted with a 5-45% acetonitrile gradient in 0.2% formic acid over a 50 min interval. Data-dependent acquisition of collision-induced dissociation MS/MS was utilized, and parent ion scans were run over the mass range *m*/*z *400 -2,000 at 8,100. For analysis of LC-MS/MS data, Mascot searches used the following parameters: 1.4 Da MS error, 0.8 Da MS/MS error, 1 potential missed cleavage, and variable oxidation (Methionine) [12].

### Protein identification

Data files from the chromatography runs were batch searched against the *M. avium *subsp. *paratuberculosis *K10 proteome database using the SEQUEST algorithm16 contained within Bioworks v3.1 software[13]. Inclusion of identified proeins was based on minimum cross-correlation coefficients (Xcorr) of 1.9, 2.2, and 3.75 for singly, doubly, and triply charged precursor ions respectively and a minimum ΔCn of 0.1 were both required for individual peptides. For false positive analysis, a decoy search was performed automatically by choosing the *Decoy *checkbox on the search form.

### Physicochemical characteristics and subcellular localization of the identified proteins

The full set of *M. avium *subsp. *paratuberculosis *K10 ORFs was downloaded from the NCBI databases, including 4399 genes. The codon adaptation indices (CAI) and hydrophilicity of the proteins were calculated with the standalone version of the software program CodonW (John Peden, http://bioweb.pasteur.fr/seqanal/interfaces/codonw.html). The TMHMM 2.0 program, based on a hidden Markov model http://www.cbs.dtu.dk/services/TMHMM/, was used to predict protein transmembrane topology[14]. The protein functional family was categorized according to the TubercuList http://genolist.pasteur.fr/TubercuList/.

## Results

### High-throughput identification of cell wall proteins with SDS-PAGE + LC-MS/MS

To avoid false-positive hits, we applied strict criteria for peptide and proteins identification. Additional file 1 shows detailed information about the identified proteins. In total, 309 unique proteins were identified, which included 101 proteins previously annotated as hypothetical or conserved hypothetical. Orthologues of the coding genes were found in *M. avium *subsp. *hominissuis *after blast searching the full genomic sequence using NCBI blast engine

### Hydrophobicity analysis of the identified cell wall proteins

**Figure 1:**
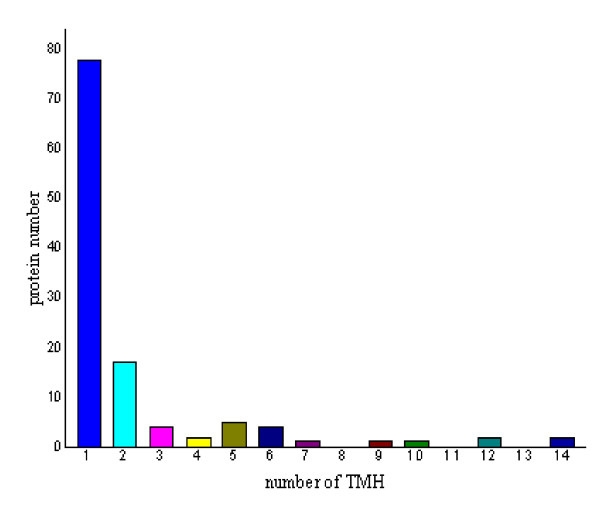
**The distribution of of Transmembrane helices (TMH) as predicted by using the TMHMM 2.0 2.0 software identified in the *Mycobacterium avium *subsp. *paratuberculosis *K10 cell wall proteome**.

Potential cell wall associated proteins with 1-15 TMHs were assigned using the software TMHMM 2.0 program against the *M. avium *subsp. *paratuberculosis *K10 protein sequence database (excluding the possible signal sequences). In our study, 120 proteins (38.83%) were identified to have at least 1 transmembrane domain. The predicted TMH numbers of these proteins ranged from 1 to 14, 18 proteins contained two TMHs and 25 proteins (8.09%) with three or more TMHs. The profile of TMH in cell wall proteins of *M. avium *subsp. *paratuberculosis *K10 is very similar to previous reports about TMH in *M. tuberculosis *cell wall proteome[15]. The distribution of these TMHs is shown in Fig. 1. Among the 309 cell wall proteins identified, it is very interesting to find that there are 157 designated as cytoplasmic, 85 proteins have an unassigned location and 67 proteins are designated as cell wall related when analyzed by PSORTb location predictions.

### Molecular mass and p*I *distributions of the identified cellwall proteins

**Figure 2:**
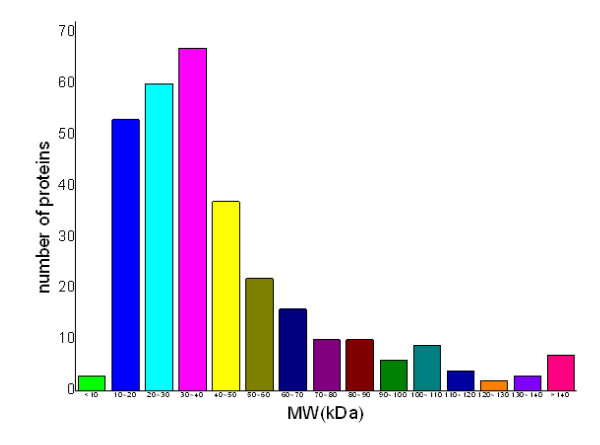
**The distribution of molecular mass (*M*_*r*_) of the identified *Mycobacterium avium *subsp. *paratuberculosis *K10 cell wall proteome**.

**Figure 3:**
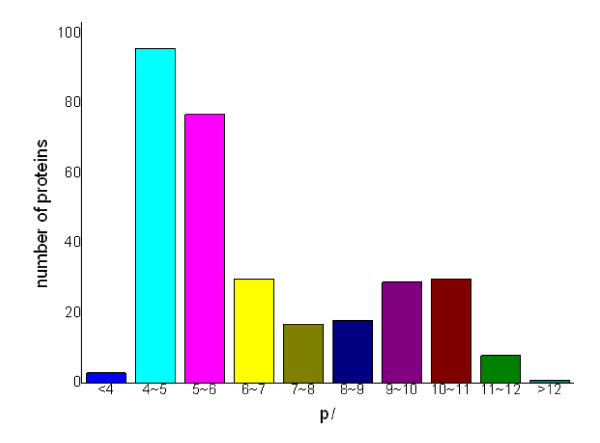
**The distribution of isoelectric points(P*I *values) of the identified *Mycobacterium avium *subsp. *paratuberculosis *K10 cell wall proteome**.

**Figure 4:**
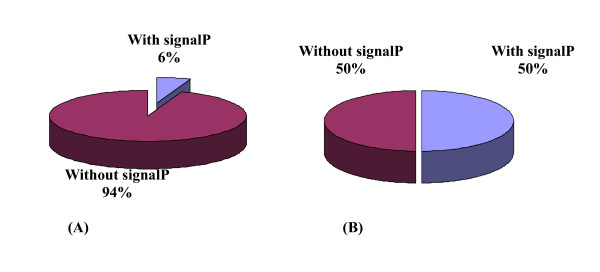
**The distribution of proteins with asignalpeptide (SP)in (A) *Mycobacterium avium *subsp. *paratuberculosis *K10 cell wall proteome; (B) *Mycobacterium avium *subsp. *paratuberculosis *K10 cell surface-exposed proteome**.

The theoretical *M*_r _distribution of the identified cell wall proteins ranged from 2.92 kDa to 683.12 kDa. Moreover, proteins between *M*_r _10 and 50 kDa were in the majority, representing approximately 58.25% (180 out of 309) of all the identified cell wall proteins. Detailed distributions are shown in Fig. 2. The theoretical p*I *scores of the identified cell wall proteins ranged from 3.77 to 12.31. Detailed distributions are shown in Fig. 3. There are 39 proteins with p*I *scores over 10 and 15 proteins with *M*_r _over 100 kDa. Taking GRAVY value into account, there will be at least 39 proteins beyond the general 2-DE separation limits. Additionally, there are 49 proteins with predicted signal peptide in the 309 identified cell wall proteins (Fig. 4A).

### Analysis of functional groups in identified cell wall protein

Based on the Pasteur Institute functional classification tree http://genolist.pasteur.fr/TubercuList/, 309 identified proteins were distributed across eight of these functional groups (See table 1 for details). Most of the identified proteins were involved in intermediary metabolism and respiration (functional class 5, 23.95%), cell wall and cell process (21.04%) and conserved hypothetical proteins (17.48%). 62.47% of proteins were involved in the three major functional categories above. Many unexpected proteins such as the ribosomal proteins were found to be cell wall associated, which were also found in cell wall by previous research [7,15]. It is probable that these proteins interact tightly with the cell wall and join in cell envelop processes and would be potential significance in vaccine studies. Overlap between cytosolic, membrane and cell wall proteins in large scale proteomic studies is not uncommon. Additional studies are necessary to investigate the proteins with multiple cellular locations.

**Table 1:** Functional classification of the identified *Mycobacterium avium *subsp. *paratuberculosis *K10 cell wall proteins according to Tubercurolist.

Class	Function	Cell surface	Cell wall
0	Virulence, detoxification, adaptation	7	17
1	Lipid metabolism	7	29
2	Information pathways	5	34
3	Cell-wall and cell processes	4	65
4	PE and PPE proteins	4	1
5	Intermediary metabolism and respiration	11	74
6	Proteins of unknown function	0	22
7	Regulatory proteins	1	13
8	Conserved hypothetical proteins	0	54

**Total**		**39**	**309**

The fatty acid components are the most energetically expensive molecules to produce, and thus the regulation of fatty acid production is very tightly controlled to match the growth rate of cells. Mycolic acids are major and specific long-chain fatty acids of the cell envelope of several important human pathogens such as *M. avium *subsp. *paratuberculosis*, *M. tuberculosis*, *M. leprae*, and *Corynebacterium diphtheriae*. Their biosynthesis is essential for mycobacterial growth and represents an attractive target for developing new antituberculous drugs. In this study, 19 proteins related to lipid metabolism were identified as cell wall associated proteins, which include CmaA1(Mycolic acid synthase), CmaA2, FadE25_2, fadD32, fadA_1, FadB_1, fadD12_1, FadE3_2, FadD6, FadE24, FadE23, FadD29, fadA2, FadE20_3, Pks13, DesA1, DesA2, DesA3_2, fabG.

It is known for many bacterial species that there are tens of proteins required for cell division, for most of which exact functions are still unknown. In this study, the proteins related to cell division, ftsH, ftsZ, ftsX, ftsE, Wag31 (a homologue of the cell division protein DivIVA), PknA/PknB were identified as cell wall related proteins in this study.

### Surface exposed proteins

**Figure 5:**
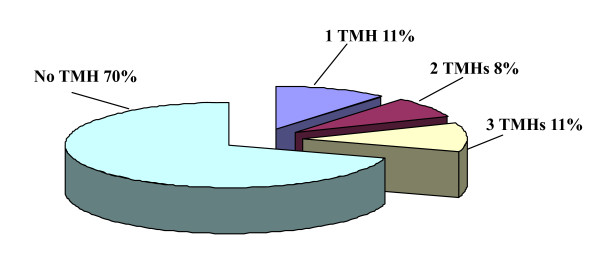
**Transmembrane helices (TMH) in the identified surface exposed proteins of *Mycobacterium avium subsp*. *paratuberculosis *K10**.

**Figure 6:**
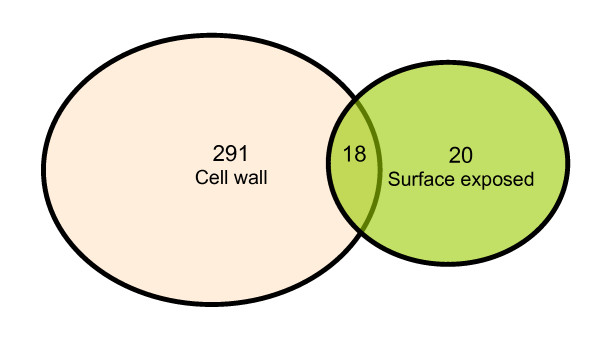
**Venn diagram showing the overlap between the identified cell wall and cell surface exposed proteins**.

The integrity of the cells after trypsin treatment was confirmed by microscopy (live/dead staining) and cultivation methods, results of which confirmed the integrity of the cells (data not shown). Peptides released into the supernatant were collected to be fully digested with trypsin for 12~14 hrs, then concentrated and analyzed by LC-MS/MS. A total of 38 cell surface exposed proteins were successfully identified (as seen in table. 2). The predicted TMH numbers of these proteins ranged from 1 to 3, and 19% of which contained at least two TMHs. The distribution of these TMHs is listed in Fig. 5. 50% of the identified proteins have signal peptides (Fig 4B). As seen from Fig. 6, 18 proteins of 38 found surface-exposed proteins overlapped with the cell wall proteins, which include 3-oxoacyl-(acyl carrier protein) synthase II, acetyl-CoA acetyltransferase, acyl carrier protein, AhpD, AtpH, chaperonin GroEL, DesA2, DNA-directed RNA polymerase subunit alpha, elongation factor Tu, FadE24, FadE3_2, FixB, hypothetical protein MAP1563c, hypothetical protein MAP3007, hypothetical protein MAP3567, S-adenosyl-L-homocysteine hydrolase, SerA and Wag31. As seen from table. 3, among the 18 proteins that were identified as both the cell wall and cell surface proteins, there are two proteins (acyl carrier protein and S-adenosyl-L-homocysteine hydrolase) which are not found in the environmental *M. smegmatis*, five proteins (acyl carrier protein, AtpH, DesA2, hypothetical protein MAP1563c and hypothetical protein MAP3567) which are not found in *Nocardia farcinica*, a pathogenic member of the Actinomycetes, and nine proteins (acyl carrier protein, AhpD, AtpH, DesA2, FadE24, hypothetical protein MAP1563c, hypothetical protein MAP3007, hypothetical protein MAP3567 and Wag31) which are not found in *Streptomyces coelicolor A3*, a soil-dwelling member of the Actinomycetes.

**Table 2:** Cell surface proteins identified by trypsin-shaving

Genbank accession	Locus tag	gene name	Tuberculosis H37Rv homologue	Functional classification	TMHs	p*I*	MW	Signal P (Y/N)	Protein name
gi|41410034	MAP3936		Rv0440	0		4.60	56635.20	N	chaperonin GroEL
gi|41410241	MAP4143	Tuf	Rv0685	2		4.96	43765.28	N	elongation factor Tu
gi|41409122	MAP3024c		Rv2986c	2		12.48	22187.24		HupB
gi|41409749	MAP3651c	FadE3_2	Rv0215c	1	1	6.12	44045.71	N	FadE3_2
gi|41408095	MAP1997	acpP	Rv2244	1		3.77	12483.25	N	acyl carrier protein
gi|33327135	MAP3968	hbhA	Rv0475	3		9.84	21534.4	Y	heparin-binding hemagglutinin adhesin-like protein
gi|41407220	MAP1122	mihF	Rv1388	2		11.02	20817.01	Y	MIHF
gi|13375557	MAP1589c	ahpC	Rv2428	0		4.28	21566.2	Y	alkylhydroperoxidase C
gi|41407604	MAP1506	PPE26	Rv1789	6		4.18	38588.55		hypothetical protein MAP1506
gi|41409460	MAP3362c	sahH	Rv3248c	7		4.72	54428.58	N	S-adenosyl-L-homocysteine hydrolase
gi|41407617	MAP1519	PPE30	Rv1802	6		5.36	46020.98	Y	hypothetical protein MAP1519
gi|41408796	MAP2698c	DesA2	Rv1094	1		4.62	31465.61	N	DesA2
gi|41408096	MAP1998	KasA	Rv2245	1	1	4.84	43740.03	N	3-oxoacyl-(acyl carrier protein) synthase II
gi|41409938	MAP3840	dnaK	Rv0350	0		4.59	66830.67	Y	molecular chaperone DnaK
gi|41410362	MAP4264	groES	Rv3418c	0		4.34	10772.12	Y	co-chaperonin GroES
gi|41409791	MAP3693	fadA2	Rv0243	1		6.19	46844.14	N	acetyl-CoA acetyltransferase
gi|41407661	MAP1563c		Rv1855c	7		4.71	33359.95	N	hypothetical protein MAP1563c
gi|41406496	MAP0398c		Rv3676	9		10.24	24759.2	Y	PROBABLE TRANSCRIPTIONAL REGULATORY PROTEIN
gi|41406994	MAP0896	sucC	Rv0951	7		4.74	40925.62	Y	succinyl-CoA synthetase subunit beta
gi|41407064	MAP0966c	PPE61	Rv3532	6		4.15	41549.19	Y	hypothetical protein MAP0966c
gi|41409131	MAP3033c	SerA	Rv2996c	7		4.6	54501.08	N	SerA
gi|41409105	MAP3007		Rv2971	7		4.27	30009.58	N	hypothetical protein MAP3007
gi|41409286	MAP3188	FadE24	Rv3139	1	1	5.60	49533.15	N	FadE24
gi|41407088	MAP0990	eno	Rv1023	7		4.26	44929.34	Y	phosphopyruvate hydratase
gi|41407686	MAP1588c	AhpD	Rv2429	0		4.73	18839.91	N	AhpD
gi|41407262	MAP1164	gap	Rv1436	7		5.05	35923.74	Y	glyceraldehyde-3-phosphate dehydrogenase
gi|41407987	MAP1889c	Wag31	Rv2145c	3		4.38	28046.25	N	Wag31
gi|41410331	MAP4233	rpoA	Rv3457c	2		4.40	37699.63	N	DNA-directed RNA polymerase subunit alpha
gi|41410265	MAP4167	rpsC	Rv0707	2		10.71	29987.79	Y	rpsC
gi|41409159	MAP3061c	fixA	Rv3029c	7		4.37	28080.71	Y	PROBABLE ELECTRON TRANSFER FLAVOPROTEIN (BETA-SUBUNIT) FIXA
gi|41408326	MAP2228	fadE18	Rv1933c	1		6.18	38477.01	Y	hypothetical protein MAP2228
gi|41410331	MAP4233	rpoA	Rv3457c	2		4.40	37699.63	N	DNA-directed RNA polymerase subunit alpha
gi|41408552	MAP2453c	AtpH	Rv1307	7		4.65	59985.27	N	AtpH
gi|41409103	MAP3005c		Rv2969c	3		5.77	26829.39	Y	hypothetical protein MAP3005c
gi|41408378	MAP2280c	clpP2	Rv2460c	0		4.76	23507.67	Y	ATP-dependent Clp protease proteolytic subunit
gi|41409665	MAP3567		Rv0148	7		5.62	30180.38	N	hypothetical protein MAP3567
gi|41407606	MAP1508	esxP	Rv2347c	3		5.02	10977.18	Y	hypothetical protein MAP1508

**Table 3:** Orthologues of the *M. avium *subsp. *paratuberculosis *proteins that were identified as both cell surface and cell wall associated in related organisms

accession	Locus tag	Protein name	Orthologues (Y/N)			
			*M. avium 104*	*M. smegmatis*	*Nocardia farcinica*	*Streptomyces coelicolor A3*
gi|41408096	MAP1998	3-oxoacyl-(acyl carrier protein) synthase II	Y	Y	Y	Y
gi|41409791	MAP3693	acetyl-CoA acetyltransferase	Y	Y	Y	Y
gi|41408095	MAP1997	acyl carrier protein	Y	N	N	N
gi|41407686	MAP1588c	AhpD	Y	Y	Y	N
gi|41408552	MAP2453c	AtpH	Y	Y	N	N
gi|41410034	MAP3936	chaperonin GroEL	Y	Y	Y	Y
gi|41408796	MAP2698c	DesA2	Y	Y	N	N
gi|41410331	MAP4233	DNA-directed RNA polymerase subunit alpha	Y	Y	Y	Y
gi|41410241	MAP4143	elongation factor Tu	Y	Y	Y	Y
gi|41409286	MAP3188	FadE24	Y	Y	Y	N
gi|41409749	MAP3651c	FadE3_2	Y	Y	Y	Y
gi|41409159	MAP3061c	FixB	Y	Y	Y	Y
gi|41407661	MAP1563c	hypothetical protein MAP1563c	Y	Y	N	N
gi|41409105	MAP3007	hypothetical protein MAP3007	Y	Y	Y	N
gi|41409665	MAP3567	hypothetical protein MAP3567	Y	Y	N	N
gi|41409460	MAP3362c	S-adenosyl-L-homocysteine hydrolase	Y	N	Y	Y
gi|41409131	MAP3033c	SerA	Y	Y	Y	Y
gi|41407987	MAP1889c	Wag31	Y	Y	Y	N

## Discussion

In this study, cell wall proteins were first separated by SDS-PAGE according to their molecular weight followed by in-gel digested with trypsin into complex peptide mixture, and then the mixture was analyzed directly by LC-MS/MS. Subsequently, protein identifications were determined by database searching software [16]. Our experiments led to the identification of a much wider range of proteins in cell wall fraction than those identified using the conventional 2-DE based method[17] and can therefore be used as a comprehensive reference profile for *Mycobacterium *spp. cell wall proteomic studies. Additionally, the surface exposed proteome was identified by an enzymatic shaving technique. Two interesting observations result from the cell wall profile. Firstly, there is a discrepancy between the identified surface exposed proteins and the complete cell wall proteome. This is likely due to the loose association of these proteins with the cell wall which makes them prone to detachment. Indeed, some surface proteins are assumed to be attached to the cell wall in a non-covalent way and have been reported to be lost during mild standard manipulations[18,19]. Secondly, some proteins are not expected to be localized in the cell wall based on their annotated function. Till now, it is still unclear how proteins such as GroEL and elongation factor TU, leaving the bacterial cell, are retained on the cell surface and whether they have an additional function when associated with the cell wall different from their known function inside the bacterial cell. EF-Tu indeed was identified as a cell wall related protein in this study and has already been identified as cell wall protein in other studies [7]. It was found that only a small percentage of the proteins identified were classified as membrane bound by PSORTb in this study. The existing methods of subcellular localization have been developed for prokaryotic proteins mainly for bacterial proteins like PSORTb, PSLpred, CELLO, LOCtree, P-classifier, Gpos-ploc, GNBSL [20,21]. Not any method could correctly predict all proteins location. One of the challenges in subcellular localization is to predict location of proteins having multiplelocation [22]. It was reported that PSORTb version 2.0 correctly predicted 88% cytoplasmic, 81% integral membrane and 80% secretory proteins. PSORTb predicted only 18% membrane-attached into cytoplasmic membrane proteins and rest of them as unknown proteins[23].

In this study, one PPE protein was identified in the cell wall fraction and four PPE proteins were identified in the cell surfaced exposed proteome. The names PE and PPE are derived from the motifs Pro-Glu and Pro-Pro-Glu, respectively, found in conserved domains near the N termini of these proteins. The PE and PPE gene families are highly expanded in the pathogenic members of this genus but show a conspicuous paucity in the nonpathogenic species. Although no precise function is known for any member of these families, members of the PE and PPE families have been linked to virulence [24,25] or have at least been shown to influence interactions with other cells [26].

It is known for many bacteria that there are tens of proteins required for cell division, most of which exact functions are still unknown. The proteins related to cell division, ftsH, ftsZ, ftsX, ftsE, Wag31 (a homologue of the cell division protein DivIVA), PknA/PknB were identified as cell wall related proteins in this study. The divIVA gene, which for the most part is confined to gram-positive bacteria, was first identified in *Bacillus subtilis*. Cells with a mutation in this gene have a reduced septation frequency and undergo aberrant polar division, leading to the formation of anucleate minicells[19,22,25]. A divIVA gene is also present in *Streptomyces coelicolor*[27] and in other actinomycetes, like *Mycobacterium tuberculosis*, where Wag31 (antigen 84) is proposed to be involved in cell shape maintenance[28]. FtsZ is a bacterial cytoskeletal protein that is essential for cell division many prokaryotes [29]. It has been shown to be a bacterial homolog of eukaryotic tubulin, based both on a low sequence identity and a striking structural similarity [30]. It appears to act at the earliest step in septation and is required through the final step of cytokinesis[31]. FtsE, in association with the integral membrane protein FtsX, is involved in the assembly of potassium ion transport proteins, both of which being relevant to the tubercle bacillus. Recently FtsE and FtsX have been found to localize to the septal ring in *E. coli*, with the localization requiring the cell division proteins FtsZ, FtsA, and ZipA but not FtsK, FtsQ, FtsL, and FtsI proteins, suggestive of a role for FtsEX in cell division. The receptor-like protein kinase PknB is encoded by the distal gene in a highly conserved operon, present in all actinobacteria, that may control cell shape and cell division. Genes coding for a PknB-like protein kinase are also found in many more distantly related gram-positive bacteria. It was demonstrated that the Ser/Thr protein kinase PknB is essential for sustaining mycobacterial growth and support the development of protein kinase inhibitors as new potential antituberculosis drugs[32].

The fatty acid components are the most energetically expensive molecules to produce, and thus the regulation of fatty acid production is very tightly controlled to match the growth rate of cells. Mycolic acids are major and specific long-chain fatty acids of the cell envelope of several important human pathogens such as *Mycobacterium avium *subsp. *paratuberculosis*, *Mycobacterium tuberculosis*, *M. leprae*, and *Corynebacterium diphtheriae*. Their biosynthesis is essential for mycobacterial growth and represents an attractive target for developing new antituberculous drugs. In this study, 19 proteins proteins related to lipid metabolism were identified as cell wall associated proteins, which include CmaA1(Mycolic acid synthase), CmaA2, FadE25_2, fadD32, fadA_1, FadB_1, fadD12_1, FadE3_2, FadD6, FadE24, FadE23, FadD29, fadA2, FadE20_3, Pks13, DesA1, DesA2, DesA3_2, fabG.

CmaA1 is a *cis *cyclopropanesynthetase which produces a distal *cis *cyclopropane ring in the alpha mycolate of *M. smegmatis *[33]. *cmaA2 *is the *trans *cyclopropane synthetase for both the methoxy and ketomycolates.

pks13 gene encodes condensase, the enzyme that performs the final condensation step of mycolic acid biosynthesis and is flanked by two genes, fadD32 and accD4, both of which have been indicated to play a role in the activation of the substrates of the condensase [34]. DesA1 is homologous to the plant stearoyl-ACP desaturase which introduce the first double bond in the saturated fatty acids, C16 and C18, the products of fatty acid biosynthesis. These fatty acids are then incorporated in the membrane glycerolipids, cuticular lipids and oilseeds of plants[35]. Involvement of these proteins in mycolic acid synthesis has been suggested based on sequence annotations[36] and structural characterization. However, experimental evidence regarding their functional roles are not presently available. FadE3_2 and FadE25_2 are enzymes involved in electron transport with acyl-CoA dehydrogenase activity. Such enzymes act at the first dehydrogenase step of the β-oxidation of fatty acids. A study of protein expression of *M. avium *engulfed by macrophages found that FadE2, a protein with 98% protein domain similarity to FadE3_2, was up-regulated[37]. It appears that these proteins are important in the utilisation of fatty acids as a carbon source and that they may have a direct correlation to mycobacterial replication, particularly within host macrophages.

A total of 18 identified proteins, HspR, DnaJ, DnaK, KatG, LprG, HtrA, PhoR, PMM, PepA, MmpL3, sdhA, ClpB, hbhA, HBHA, Tuf, groES, manB, DesA3_2, can be considered putative virulence factors as they have previously been suggested to play some role in virulence [38-52].

## Conclusions

We have obtained a comprehensive picture of the *M. avium *subsp. *paratuberculosis *K10 cell wall protein repertoire, with an additional insight in the portion of these proteins that are cell surface exposed. With 309 distinct proteins identified, this study represents the first proteomic analysis of cell wall proteins of *M. avium *subsp. *paratuberculosis *K10. To our knowledge, this is also the first report of a SDS-PAGE-LC-MS/MS based proteomic approach, supported with cell surface enzymatic digestion, to localize proteins in the mycobacterial cell wall. Many of the cell wall-associated proteins found in this study are involved in cell division, lipid metabolism or are putative virulence factors. Therefore, they should be considered as new potential antigens for vaccine development to prevent *M. avium *subsp. *paratuberculosis *K10 infection.

## Competing interests

The authors declare that they have no competing interests.

## Authors' contributions

ZH carried out the experiments, participated in the data analysis and drafted the manuscript. JDB conceived of the study, and participated in its design and coordination. All authors read and approved the final manuscript.

## Supplementary Material

Additional file 1 **summarization of the identified cell wall proteins of *M. avium *subsp. *paratuberculosis***. The data provided summarization of the identified cell wall proteins of *M. avium *subsp. *paratuberculosis*. Click here for file
