# Supplementary material for: Localization of proteins in the cell wall of Mycobacterium avium subsp. paratuberculosis K10 by proteomic analysis
Source: Proteome Sci. 2010 Apr 8;8:21. doi: 10.1186/1477-5956-8-21 (PMC2859856; doi:10.1186/1477-5956-8-21)
Supplement: Additional file 1 — summarization of the identified cell wall proteins of M. avium subsp. paratuberculosis. The data provided summarization of the identified cell wall proteins of M. avium subsp. paratuberculosis. [file 1477-5956-8-21-S1.PDF]

Genbank accession	Locus	gene name	Function	Functional Classification	number tmd	*pI*	MW	Signal_Details	proteins
gi|41406103	MAP0005	gyrB	DNA gyrase subunit B	2		6.037920952	74521.89063	No signal peptide detected	DNA gyrase subunit B [Mycobacterium avium subsp. paratuberculosis K-10]
gi|41406108	MAP0010c		no	11				No signal peptide detected	hypothetical protein MAP0010c [Mycobacterium avium subsp. paratuberculosis K-10]
gi|41406114	MAP0016c	PknB		9	1	5.219321251	66382.375	No signal peptide detected	PknB [Mycobacterium avium subsp. paratuberculosis K-10]
gi|41406116	MAP0018c	PknA		9		7.459609985	27282.02148	No signal peptide detected	PknA [Mycobacterium avium subsp. paratuberculosis K-10]
gi|41406119	MAP0021c	Ppp	serine/threonine phosphatase	9	1	4.65501833	52659.69141	No signal peptide detected	Ppp [Mycobacterium avium subsp. paratuberculosis K-10]
gi|41406132	MAP0034		low	5	2	10.13979626	44019.15234	No signal peptide detected	hypothetical protein MAP0034 transposase [Mycobacterium avium subsp. paratuberculosis K-10]
gi|41406158	MAP0060c	ino1		7	1	4.617818832	40207.52344	No signal peptide detected	hypothetical protein MAP0060c [Mycobacterium avium subsp. paratuberculosis K-10]
gi|41406162	MAP0064	PonA_1		3	2	6.625116348	91666.5	No signal peptide detected	PonA_1 [Mycobacterium avium subsp. paratuberculosis K-10]
gi|41406170	MAP0072c		low	3		10.52024651	32756.57813	No signal peptide detected	hypothetical protein MAP0072c [Mycobacterium avium subsp. paratuberculosis K-10]
gi|41406171	MAP0073c			10	1	7.447013855	28878.66211	No signal peptide detected	hypothetical protein MAP0073c [Mycobacterium avium subsp. paratuberculosis K-10]
gi|41406200	MAP0102		no	11		9.561676025	18308.47656	No signal peptide detected	hypothetical protein MAP0102 [Mycobacterium avium subsp. paratuberculosis K-10]
gi|41406201	MAP0103c		no	11		10.53604126	20179.65625	No signal peptide detected	hypothetical protein MAP0103c [Mycobacterium avium subsp. paratuberculosis K-10]
gi|41406233	MAP0135	CmaA1	Mycolic acid synthase	1	1	5.016605377	33524.91797	No signal peptide detected	CmaA1 Mycolic acid synthase [Mycobacterium avium subsp. paratuberculosis K-10]
gi|41406242	MAP0144	cysA1	low	3		9.043498993	28284.07813	No signal peptide detected	hypothetical protein MAP0144 [Mycobacterium avium subsp. paratuberculosis K-10]
gi|41406248	MAP0150c	FadE25_2	acyl-CoA dehydrogenase FADE25	1	1	5.426463127	43749.29297	No signal peptide detected	FadE25_2 [Mycobacterium avium subsp. paratuberculosis K-10]
gi|41406265	MAP0167		BXX/CFQX family protein	10	1	5.045932293	67252.96875	No signal peptide detected	hypothetical protein MAP0167 [Mycobacterium avium subsp. paratuberculosis K-10]
gi|41406282	MAP0184c			10		11.9066267	24699.86133	No signal peptide detected	hypothetical protein MAP0184c [Mycobacterium avium subsp. paratuberculosis K-10]
gi|41406310	MAP0212	glfT	bifunctional UDP-galactofuranosyl transferase GLFT	3		6.608887672	70373.8125	No signal peptide detected	hypothetical protein MAP0212 [Mycobacterium avium subsp. paratuberculosis K-10]
gi|41406311	MAP0213		transmembrane protein	3	1	5.529296398	69311.65625	No signal peptide detected	hypothetical protein MAP0213 [Mycobacterium avium subsp. paratuberculosis K-10]
gi|41406317	MAP0219	fadD32	acyl-CoA synthetase	1	3	4.559403419	191053.4375	No signal peptide detected	acyl-CoA synthetase [Mycobacterium avium subsp. paratuberculosis K-10]
gi|41406318	MAP0220	Pks13		1	1	9.209852219	110620.1953	No signal peptide detected	Pks13 [Mycobacterium avium subsp. paratuberculosis K-10]
gi|41406326	MAP0228c	EmbB		3		9.433036804	110706.8906	Signal peptide detected	EmbB [Mycobacterium avium subsp. paratuberculosis K-10]
gi|41406330	MAP0232c	EmbC		3		9.433036804	110706.8906	No signal peptide detected	EmbC [Mycobacterium avium subsp. paratuberculosis K-10]
gi|41406332	MAP0234c		short chain dehydrogenase	7	1	7.262535095	27345.33398	No signal peptide detected	short chain dehydrogenase [Mycobacterium avium subsp. paratuberculosis K-10]
gi|41406452	MAP0354c		no	11	1	6.77728653	25419.34766	No signal peptide detected	hypothetical protein MAP0354c [Mycobacterium avium subsp. paratuberculosis K-10]

gi|41406457	MAP0359c	MoxR2		9	1	5.193025589	34990.97656	No signal peptide detected	MoxR2 [Mycobacterium avium subsp. paratuberculosis K-10]
gi|41406477	MAP0379c	RsbR		9	1	4.419150829	33022.41406	No signal peptide detected	RsbR [Mycobacterium avium subsp. paratuberculosis K-10]
gi|41406493	MAP0395c		anion transporter ATPase	3		4.963462353	35918.87891	No signal peptide detected	hypothetical protein MAP0395c [Mycobacterium avium subsp. paratuberculosis K-10]
gi|41406496	MAP0398c		CRP/FNR family transcriptional regulator	9		10.02773285	24792.35156	No signal peptide detected	hypothetical protein MAP0398c [Mycobacterium avium subsp. paratuberculosis K-10]
gi|41406512	MAP0414c			0	2	6.060100555	30820.38477	No signal peptide detected	hypothetical protein MAP0414c [Mycobacterium avium subsp. paratuberculosis K-10]
gi|41406546	MAP0448	FtsH	membrane-bound protease FTSH	3	2	5.763508797	86086.76563	No signal peptide detected	FtsH [Mycobacterium avium subsp. paratuberculosis K-10]
gi|41406552	MAP0454		transmembrane protein	3	1	5.039993763	46241.39063	No signal peptide detected	hypothetical protein MAP0454 [Mycobacterium avium subsp. paratuberculosis K-10]
gi|41406558	MAP0460	Lsr2		10		10.41729546	12135.98242	No signal peptide detected	Lsr2 [Mycobacterium avium subsp. paratuberculosis K-10]
gi|41406559	MAP0461	ClpC		7		5.440739155	93268.67188	No signal peptide detected	ClpC [Mycobacterium avium subsp. paratuberculosis K-10]
gi|41406572	MAP0474c	LpqE		3		7.014480591	15704.82031	No signal peptide detected	LpqE [Mycobacterium avium subsp. paratuberculosis K-10]
gi|41406592	MAP0494		low	10		5.80034256	32610.82617	No signal peptide detected	hypothetical protein MAP0494 [Mycobacterium avium subsp. paratuberculosis K-10]
gi|41406682	MAP0584	lpqH	19 kDa lipoprotein antigen precursor LPQH [	3	1	4.170358658	12055.60352	No signal peptide detected	hypothetical protein MAP0584 [Mycobacterium avium subsp. paratuberculosis K-10]
gi|41406690	MAP0592	PhoR		8	1	5.702474594	47253.26563	No signal peptide detected	PhoR [Mycobacterium avium subsp. paratuberculosis K-10]
gi|41406712	MAP0614	hemH	phosphoribosylaminoimidazole-succinocarboxamide synthase	7		4.651627541	32913.23047	No signal peptide detected	phosphoribosylaminoimidazole-succinocarboxamide synthase [Mycobacterium avium subsp. paratuberculosis K-10]
gi|41406747	MAP0649		transcriptional regulatory protein	9		4.822549343	28003.14844	No signal peptide detected	hypothetical protein MAP0649 [Mycobacterium avium subsp. paratuberculosis K-10]
gi|41406752	MAP0654	PhoT		3		5.328458786	28215.78711	No signal peptide detected	PhoT [Mycobacterium avium subsp. paratuberculosis K-10]
gi|41406753	MAP0655c	PhoY2_2		3		4.923473358	24440.82227	No signal peptide detected	PhoY2_2 [Mycobacterium avium subsp. paratuberculosis K-10]
gi|41406756	MAP0658c	DesA1		1		5.883097649	39218.87109	No signal peptide detected	DesA1 [Mycobacterium avium subsp. paratuberculosis K-10]
gi|41406810	MAP0712c		low	9		6.238975525	23982.45703	No signal peptide detected	hypothetical protein MAP0712c [Mycobacterium avium subsp. paratuberculosis K-10]
gi|41406848	MAP0750c		mce ?	3	1	7.003967285	14682.72754	No signal peptide detected	hypothetical protein MAP0750c [Mycobacterium avium subsp. paratuberculosis K-10]
gi|41406849	MAP0751c		mce ?	3	1	4.420849323	31874.71094	No signal peptide detected	hypothetical protein MAP0751c [Mycobacterium avium subsp. paratuberculosis K-10]
gi|41406887	MAP0789	fadA_1		1		4.774514198	42590.97656	No signal peptide detected	acetyl-CoA acetyltransferase [Mycobacterium avium subsp. paratuberculosis K-10]
gi|41406888	MAP0790	FadB_1		1	1	5.246280193	76168.60156	No signal peptide detected	FadB_1 [Mycobacterium avium subsp. paratuberculosis K-10]
gi|41406912	MAP0814c			3	1	8.195690155	18110.76953	No signal peptide detected	hypothetical protein MAP0814c [Mycobacterium avium subsp. paratuberculosis K-10]
gi|41406915	MAP0817c		no	11		10.13368416	17579.0625	No signal peptide detected	hypothetical protein MAP0817c [Mycobacterium avium subsp. paratuberculosis K-10]
gi|41406924	MAP0826c			10		4.323260307	16640.81836	No signal peptide detected	hypothetical protein MAP0826c [Mycobacterium avium subsp. paratuberculosis K-10]
gi|41406932	MAP0834c	prrA	two component response transcriptional regulatory	9		4.686465263	25021.5	No signal peptide detected	hypothetical protein MAP0834c [Mycobacterium avium subsp. paratuberculosis K-10]

			protein PRRA						
gi|41406943	MAP0845			10	5	10.16833591	31454.12695	No signal peptide detected	hypothetical protein MAP0845 [Mycobacterium avium subsp. paratuberculosis K-10]
gi|41406970	MAP0872	Pst3		3	1	5.771232605	34245.64844	Signal peptide detected	PhoS2_2 [Mycobacterium avium subsp. paratuberculosis K-10]
gi|41406985	MAP0887c		low	10	1	10.29195976	35534.90234	No signal peptide detected	hypothetical protein MAP0887c [Mycobacterium avium subsp. paratuberculosis K-10]
gi|41406991	MAP0893	UvrD		2		4.999180317	85293.59375	No signal peptide detected	UvrD [Mycobacterium avium subsp. paratuberculosis K-10]
gi|41407018	MAP0920c	mscL	large-conductance mechanosensitive channel	3	2	5.114398956	16097.50879	No signal peptide detected	large-conductance mechanosensitive channel [Mycobacterium avium subsp. paratuberculosis K-10]
gi|41407050	MAP0952		no	11	1	9.699167252	47189.05469	Signal peptide detected	hypothetical protein MAP0952 [Mycobacterium avium subsp. paratuberculosis K-10]
gi|41407060	MAP0962c		no	11		9.851275444	65496.57031	No signal peptide detected	hypothetical protein MAP0962c [Mycobacterium avium subsp. paratuberculosis K-10]
gi|41407064	MAP0966c	PPE		6		5.459999084	34884.95703	No signal peptide detected	hypothetical protein MAP0966c [Mycobacterium avium subsp. paratuberculosis K-10]
gi|41407066	MAP0968		no	11		5.748209953	26737.91797	No signal peptide detected	hypothetical protein MAP0968 [Mycobacterium avium subsp. paratuberculosis K-10]
gi|41407068	MAP0970		no	11		5.274540901	67995.26563	No signal peptide detected	hypothetical protein MAP0970 [Mycobacterium avium subsp. paratuberculosis K-10]
gi|41407078	MAP0980c		short chain dehydrogenase	7	1	10.48441696	31035.43945	No signal peptide detected	hypothetical protein MAP0980c [Mycobacterium avium subsp. paratuberculosis K-10]
gi|41407135	MAP1037	ruvA		2		7.339797974	20299.74805	No signal peptide detected	Holliday junction DNA helicase motor protein [Mycobacterium avium subsp. paratuberculosis K-10]
gi|41407140	MAP1042	YajC	preprotein translocase subunit YajC	3	1	4.098247051	11879.95508	No signal peptide detected	preprotein translocase subunit YajC [Mycobacterium avium subsp. paratuberculosis K-10]
gi|41407142	MAP1044	SecF	preprotein translocase subunit SecF	3	6	10.04667664	46182.96875	No signal peptide detected	preprotein translocase subunit SecF [Mycobacterium avium subsp. paratuberculosis K-10]
gi|41407148	MAP1050c	PpiB		2		5.450031281	25876.55664	No signal peptide detected	PpiB [Mycobacterium avium subsp. paratuberculosis K-10]
gi|41407182	MAP1084c		no	11	1	8.733406067	33053.35547	No signal peptide detected	hypothetical protein MAP1084c [Mycobacterium avium subsp. paratuberculosis K-10]
gi|41407193	MAP1095c		transmembrane protein	3	2	4.288509369	17024.86523	No signal peptide detected	hypothetical protein MAP1095c [Mycobacterium avium subsp. paratuberculosis K-10]
gi|41407236	MAP1138c	LprG		3	1	5.442146301	21093.85547	No signal peptide detected	LprG [Mycobacterium avium subsp. paratuberculosis K-10]
gi|41407257	MAP1159c	fadD12_1	acyl-CoA synthetase	1	1	6.294963837	58358.40625	No signal peptide detected	acyl-CoA synthetase [Mycobacterium avium subsp. paratuberculosis K-10]
gi|41407262	MAP1164	gap	glyceraldehyde-3-phosphate dehydrogenase	7		4.765625	36090.50391	No signal peptide detected	glyceraldehyde-3-phosphate dehydrogenase [Mycobacterium avium subsp. paratuberculosis K-10]
gi|41407300	MAP1202			3		4.730489731	15241.26758	No signal peptide detected	hypothetical protein MAP1202 [Mycobacterium avium subsp. paratuberculosis K-10]
gi|41407303	MAP1205	MoxR		9		5.842730522	40724.80859	No signal peptide detected	MoxR [Mycobacterium avium subsp. paratuberculosis K-10]
gi|41407305	MAP1207			3	5	9.798485756	35884.26172	No signal peptide detected	hypothetical protein MAP1207 [Mycobacterium avium subsp. paratuberculosis K-10]
gi|41407331	MAP1233			7		5.208572388	26349.73438	No signal peptide detected	hypothetical protein MAP1233 [Mycobacterium avium subsp. paratuberculosis K-10]
gi|41407332	MAP1234		glycosyl transferase	7	1	7.180412292	30191.89453	No signal peptide detected	hypothetical protein MAP1234 [Mycobacterium avium subsp. paratuberculosis K-10]
gi|41407406	MAP1308	lgt	prolipoprotein diacylglyceryl transferase	3	3	4.241112709	46282	No signal peptide detected	prolipoprotein diacylglyceryl transferase [Mycobacterium avium subsp. paratuberculosis K-10]

gi|41407413	MAP1315c	CydB		7	9	8.716197968	37876.24219	No signal peptide detected	CydB [Mycobacterium avium subsp. paratuberculosis K-10]
gi|41407414	MAP1316c	AppC		7	1	8.181507111	50046.125	No signal peptide detected	AppC [Mycobacterium avium subsp. paratuberculosis K-10]
gi|41407415	MAP1317c			3	1	10.74144745	15218.47461	No signal peptide detected	hypothetical protein MAP1317c [Mycobacterium avium subsp. paratuberculosis K-10]
gi|41407420	MAP1322	polA	DNA polymerase I	2		4.733288765	100267.6172	No signal peptide detected	DNA polymerase I [Mycobacterium avium subsp. paratuberculosis K-10]
gi|41407423	MAP1325	rpsA	30S ribosomal protein S1	2		4.61478138	53015.01563	No signal peptide detected	30S ribosomal protein S1 [Mycobacterium avium subsp. paratuberculosis K-10]
gi|41407437	MAP1339			10		5.719806671	15435.49609	No signal peptide detected	hypothetical protein MAP1339 [Mycobacterium avium subsp. paratuberculosis K-10]
gi|41407439	MAP1341	uvrA	excinuclease ABC subunit A	10		6.616667271	106498.2266	No signal peptide detected	excinuclease ABC subunit A [Mycobacterium avium subsp. paratuberculosis K-10]
gi|41407449	MAP1351c	LysX	lysyl-tRNA synthetase	2	7	8.558386803	129130.7109	No signal peptide detected	lysyl-tRNA synthetase [Mycobacterium avium subsp. paratuberculosis K-10]
gi|41407452	MAP1354		50S ribosomal protein L20	2		11.25481415	14657.08594	No signal peptide detected	50S ribosomal protein L20 [Mycobacterium avium subsp. paratuberculosis K-10]
gi|41407465	MAP1367	argG	argininosuccinate synthase	7		5.015563965	43762.88672	No signal peptide detected	argininosuccinate synthase [Mycobacterium avium subsp. paratuberculosis K-10]
gi|41407503	MAP1405			10	1	4.873733521	29036.38867	Signal peptide detected	hypothetical protein MAP1405 [Mycobacterium avium subsp. paratuberculosis K-10]
gi|41407508	MAP1410		putative initiation inhibitor protein	3		6.08882618	34179.98047	No signal peptide detected	hypothetical protein MAP1410 [Mycobacterium avium subsp. paratuberculosis K-10]
gi|41407518	MAP1420	nrp	peptide synthetase	1	4	4.688233852	683115.625	No signal peptide detected	hypothetical protein MAP1420 [Mycobacterium avium subsp. paratuberculosis K-10]
gi|41407571	MAP1473c		no	11		4.244360924	15341.00781	No signal peptide detected	hypothetical protein MAP1473c [Mycobacterium avium subsp. paratuberculosis K-10]
gi|41407600	MAP1502			10	1	4.946566105	146045.7031	No signal peptide detected	hypothetical protein MAP1502 [Mycobacterium avium subsp. paratuberculosis K-10]
gi|41407607	MAP1509			10		4.531728745	32301.29492	No signal peptide detected	hypothetical protein MAP1509 [Mycobacterium avium subsp. paratuberculosis K-10]
gi|41407608	MAP1510			3	10	9.823256493	53296.50781	No signal peptide detected	hypothetical protein MAP1510 [Mycobacterium avium subsp. paratuberculosis K-10]
gi|41407610	MAP1512			10	3	8.333826065	44151.98047	No signal peptide detected	hypothetical protein MAP1512 [Mycobacterium avium subsp. paratuberculosis K-10]
gi|41407611	MAP1513			2		4.827155113	63755.35938	No signal peptide detected	hypothetical protein MAP1513 [Mycobacterium avium subsp. paratuberculosis K-10]
gi|41407640	MAP1542			10		4.127375603	18029.07422	No signal peptide detected	hypothetical protein MAP1542 [Mycobacterium avium subsp. paratuberculosis K-10]
gi|41407646	MAP1548c			10	2	4.504608631	70788.83594	No signal peptide detected	hypothetical protein MAP1548c [Mycobacterium avium subsp. paratuberculosis K-10]
gi|41407661	MAP1563c		oxidoreductase	7		4.711359978	33359.95703	No signal peptide detected	hypothetical protein MAP1563c [Mycobacterium avium subsp. paratuberculosis K-10]
gi|41407668	MAP1570		no	11	2	12.30872345	2924.314941	No signal peptide detected	hypothetical protein MAP1570 [Mycobacterium avium subsp. paratuberculosis K-10]
gi|41396026	MAP1575c	uppP	undecaprenyl pyrophosphate phosphatase	3	5	10.66951561	36608.58594	No signal peptide detected	hypothetical protein MAP_1575c [Mycobacterium avium subsp. paratuberculosis K-10]
gi|41407681	MAP1583c			10				No signal peptide detected	hypothetical protein MAP1583c [Mycobacterium avium subsp. paratuberculosis K-10]
gi|41407686	MAP1588c	AhpD		0		4.737841606	18839.91992	No signal peptide detected	AhpD [Mycobacterium avium subsp. paratuberculosis K-10]
gi|41407687	MAP1589c	AhpC		0		4.190455437	21638.66992	No signal peptide detected	AhpC [Mycobacterium avium subsp. paratuberculosis K-10]
gi|41407694	MAP1596		integral membrane protein	3	1	9.25302124	67704.88281	No signal peptide detected	hypothetical protein MAP1596 [Mycobacterium avium subsp. paratuberculosis K-10]

gi|41407695	MAP1597			10		4.616318703	18341.25195	No signal peptide detected	hypothetical protein MAP1597 [Mycobacterium avium subsp. paratuberculosis K-10]
gi|41407704	MAP1606c			10		8.34312439	17763.23828	No signal peptide detected	hypothetical protein MAP1606c [Mycobacterium avium subsp. paratuberculosis K-10]
gi|41407707	MAP1609c	FbpB		1	1	4.742118835	30800.36719	Signal peptide detected	FbpB [Mycobacterium avium subsp. paratuberculosis K-10]
gi|41407741	MAP1643	aceAb	isocitrate lyase	7		5.153935432	85202.92969	No signal peptide detected	isocitrate lyase [Mycobacterium avium subsp. paratuberculosis K-10]
gi|41407744	MAP1646c			10		10.45363331	16681.98438	No signal peptide detected	hypothetical protein MAP1646c [Mycobacterium avium subsp. paratuberculosis K-10]
gi|41407751	MAP1653	tpx	thiol peroxidase	0		4.077660561	16682.62695	No signal peptide detected	thiol peroxidase [Mycobacterium avium subsp. paratuberculosis K-10]
gi|41407766	MAP1668c	KatG		0		4.705861568	81740.17188	No signal peptide detected	KatG [Mycobacterium avium subsp. paratuberculosis K-10]
gi|41407819	MAP1721c		no	11		10.25370693	24216.50586	No signal peptide detected	hypothetical protein MAP1721c [Mycobacterium avium subsp. paratuberculosis K-10]
gi|41407840	MAP1742c			10	2	5.825857162	30563.17773	No signal peptide detected	hypothetical protein MAP1742c [Mycobacterium avium subsp. paratuberculosis K-10]
gi|41407842	MAP1744		no	11		9.076328278	12663.44922	No signal peptide detected	hypothetical protein MAP1744 [Mycobacterium avium subsp. paratuberculosis K-10]
gi|41407844	MAP1746c			10	1	5.890982628	36977.36328	No signal peptide detected	hypothetical protein MAP1746c [Mycobacterium avium subsp. paratuberculosis K-10]
gi|41407852	MAP1754c			10		5.672112465	30974.54297	No signal peptide detected	hypothetical protein MAP1754c [Mycobacterium avium subsp. paratuberculosis K-10]
gi|41407897	MAP1799c	ppm1	polyprenol-monophospho mannose synthase Ppm1	3		7.902557373	29417.64258	No signal peptide detected	hypothetical protein MAP1799c [Mycobacterium avium subsp. paratuberculosis K-10]
gi|41407901	MAP1803			10		7.131069183	24844.39844	No signal peptide detected	hypothetical protein MAP1803 [Mycobacterium avium subsp. paratuberculosis K-10]
gi|41407902	MAP1804c			10		10.44062805	13783.4502	No signal peptide detected	hypothetical protein MAP1804c [Mycobacterium avium subsp. paratuberculosis K-10]
gi|41407935	MAP1837c			10		5.226226807	54632.83594	No signal peptide detected	hypothetical protein MAP1837c [Mycobacterium avium subsp. paratuberculosis K-10]
gi|41407987	MAP1889c	Wag31		3		4.380401611	28046.25	No signal peptide detected	Wag31 [Mycobacterium avium subsp. paratuberculosis K-10]
gi|41407992	MAP1894c	FtsZ	cell division protein FtsZ	3		4.312954903	39420.41406	No signal peptide detected	cell division protein FtsZ [Mycobacterium avium subsp. paratuberculosis K-10]
gi|41408004	MAP1906c	MraZ		10		5.369321823	15854.68652	No signal peptide detected	hypothetical protein MAP1906c [Mycobacterium avium subsp. paratuberculosis K-10]
gi|41408007	MAP1909	LppM		3	2	10.04708576	25989.44336	No signal peptide detected	LppM [Mycobacterium avium subsp. paratuberculosis K-10]
gi|41408013	MAP1915			10		8.345430374	52857.88672	No signal peptide detected	hypothetical protein MAP1915 [Mycobacterium avium subsp. paratuberculosis K-10]
gi|41408032	MAP1934	QcrA		7	3	7.76216507	46305.20313	No signal peptide detected	QcrA [Mycobacterium avium subsp. paratuberculosis K-10]
gi|41408038	MAP1940c	CtaC		7	1	5.981622696	34445.20313	No signal peptide detected	CtaC [Mycobacterium avium subsp. paratuberculosis K-10]
gi|41408041	MAP1943			3	1	8.868839264	23661.77539	No signal peptide detected	hypothetical protein MAP1943 [Mycobacterium avium subsp. paratuberculosis K-10]
gi|41408043	MAP1945c			10		4.618475914	38743.95313	No signal peptide detected	hypothetical protein MAP1945c [Mycobacterium avium subsp. paratuberculosis K-10]
gi|41408054	MAP1956	dlaT	dihydrolipoamide acetyltransferase	7		4.480209827	61154.69922	No signal peptide detected	dihydrolipoamide acetyltransferase [Mycobacterium avium subsp. paratuberculosis K-10]
gi|41408055	MAP1957			10	1	6.606891632	29655.90039	Signal peptide detected	hypothetical protein MAP1957 [Mycobacterium avium subsp. paratuberculosis K-10]
gi|41408063	MAP1965c	GlnE		7		6.078052521	108588.5234	No signal peptide detected	GlnE [Mycobacterium avium subsp. paratuberculosis K-10]
gi|41408064	MAP1966c	GlnA2		7	1	5.04861021	49588.55078	No signal peptide detected	GlnA2 [Mycobacterium avium subsp. paratuberculosis K-10]

gi|41408092	MAP1994	AceE	pyruvate dehydrogenase subunit E1	7		5.341408253	103361.8672	No signal peptide detected	pyruvate dehydrogenase subunit E1 [Mycobacterium avium subsp. paratuberculosis K-10]
gi|41408095	MAP1997	acpP	acyl carrier protein	1		3.775923729	12483.25684	No signal peptide detected	acyl carrier protein [Mycobacterium avium subsp. paratuberculosis K-10]
gi|41408096	MAP1998	KasA	3-oxoacyl-(acyl carrier protein) synthase II	1	1	4.84876442	43740.03516	No signal peptide detected	3-oxoacyl-(acyl carrier protein) synthase II [Mycobacterium avium subsp. paratuberculosis K-10]
gi|41408097	MAP1999	KasB	3-oxoacyl-(acyl carrier protein) synthase II	1		5.507079601	46400.86328	No signal peptide detected	3-oxoacyl-(acyl carrier protein) synthase II [Mycobacterium avium subsp. paratuberculosis K-10]
gi|41408155	MAP2057		haloalkane dehalogenase	7		9.022546768	33837.5625	No signal peptide detected	haloalkane dehalogenase [Mycobacterium avium subsp. paratuberculosis K-10]
gi|41408167	MAP2069	htpG	heat shock protein 90	0		4.585990906	72818.79688	No signal peptide detected	heat shock protein 90 [Mycobacterium avium subsp. paratuberculosis K-10]
gi|41408168	MAP2070			10		8.219444275	22305.02148	No signal peptide detected	hypothetical protein MAP2070 [Mycobacterium avium subsp. paratuberculosis K-10]
gi|41408173	MAP2075c		low	7		8.209774017	23840.28906	No signal peptide detected	hypothetical protein MAP2075c [Mycobacterium avium subsp. paratuberculosis K-10]
gi|41408229	MAP2131c	SodC	periplasmic superoxide dismutase [Cu-Zn]	0	1	5.511400223	17307.87891	Signal peptide detected	hypothetical protein MAP2131c [Mycobacterium avium subsp. paratuberculosis K-10]
gi|41408236	MAP2138	ArsR		9		6.024642944	14655.0127	No signal peptide detected	hypothetical protein MAP2138 [Mycobacterium avium subsp. paratuberculosis K-10]
gi|41408241	MAP2143	amiA2	amidase	7	1	8.039031982	52190.53516	No signal peptide detected	amidase [Mycobacterium avium subsp. paratuberculosis K-10]
gi|41408251	MAP2153		no	11	1	5.274528503	39343.26172	No signal peptide detected	hypothetical protein MAP2153 [Mycobacterium avium subsp. paratuberculosis K-10]
gi|41408261	MAP2163c	hrcA	heat-inducible transcription repressor	0		5.023337364	36827.38281	No signal peptide detected	heat-inducible transcription repressor [Mycobacterium avium subsp. paratuberculosis K-10]
gi|41408308	MAP2210c	CysA		3		6.435405731	38808.25781	No signal peptide detected	CysA [Mycobacterium avium subsp. paratuberculosis K-10]
gi|41408311	MAP2213c	SubI		3	1	6.616462708	32896.94531	Signal peptide detected	SubI [Mycobacterium avium subsp. paratuberculosis K-10]
gi|41408328	MAP2230c	pks12	polyketide synthase	1	6	4.932015896	391467.8125	No signal peptide detected	hypothetical protein MAP2230c [Mycobacterium avium subsp. paratuberculosis K-10]
gi|41408337	MAP2239	MmpL4_4		3	12	6.325704098	105726.5234	No signal peptide detected	MmpL4_4 [Mycobacterium avium subsp. paratuberculosis K-10]
gi|41408368	MAP2270c	FolC		7	2	4.435044765	50777.95703	No signal peptide detected	FolC [Mycobacterium avium subsp. paratuberculosis K-10]
gi|41408370	MAP2272c			10	1	7.062286377	44517.66406	No signal peptide detected	hypothetical protein MAP2272c [Mycobacterium avium subsp. paratuberculosis K-10]
gi|41408373	MAP2275c	MobA	molybdopterin-guanine dinucleotide biosynthesis protein A	7		5.256031036	21064.66406	No signal peptide detected	molybdopterin-guanine dinucleotide biosynthesis protein A [Mycobacterium avium subsp. paratuberculosis K-10]
gi|41408375	MAP2277c			7	2	4.876101494	69381.67969	No signal peptide detected	hypothetical protein MAP2277c [Mycobacterium avium subsp. paratuberculosis K-10]
gi|41408386	MAP2288c			10		4.884742737	16264.41992	No signal peptide detected	hypothetical protein MAP2288c [Mycobacterium avium subsp. paratuberculosis K-10]
gi|41408393	MAP2295c		putative ABC transporter ATP-binding protein	3		4.877933502	62011.41016	No signal peptide detected	putative ABC transporter ATP-binding protein [Mycobacterium avium subsp. paratuberculosis K-10]
gi|41408416	MAP2318		short-chain type dehydrogenase/reductase	7	1	7.263031006	27916.50391	No signal peptide detected	hypothetical protein MAP2318 [Mycobacterium avium subsp. paratuberculosis K-10]
gi|41408533	MAP2435c		thioredoxin	7		3.991657257	28563.23438	Signal peptide detected	hypothetical protein MAP2435c [Mycobacterium avium subsp. paratuberculosis K-10]
gi|41408538	MAP2440		adenylate cyclase	7	6	8.407072067	58874.37109	No signal peptide detected	hypothetical protein MAP2440 [Mycobacterium avium subsp. paratuberculosis K-10]
gi|41408548	MAP2450c		F0F1 ATP synthase subunit epsilon	7		4.136866093	13122.04785	No signal peptide detected	F0F1 ATP synthase subunit epsilon [Mycobacterium avium subsp. paratuberculosis K-10]

gi|41408550	MAP2451c		F0F1 ATP synthase subunit gamma	7		4.648453712	44281.76172	No signal peptide detected	F0F1 ATP synthase subunit gamma [Mycobacterium avium subsp. paratuberculosis K-10]
gi|41408551	MAP2452c		F0F1 ATP synthase subunit alpha	7		5.078123093	33572.74609	No signal peptide detected	F0F1 ATP synthase subunit alpha [Mycobacterium avium subsp. paratuberculosis K-10]
gi|41408552	MAP2453c	AtpH		7		4.65002203	59985.27344	No signal peptide detected	AtpH [Mycobacterium avium subsp. paratuberculosis K-10]
gi|41408553	MAP2454c		F0F1 ATP synthase subunit B	7	1	4.939547062	48531.42578	No signal peptide detected	F0F1 ATP synthase subunit B [Mycobacterium avium subsp. paratuberculosis K-10]
gi|41408555	MAP2456c		F0F1 ATP synthase subunit A	7	1	4.418239594	6018.287598	No signal peptide detected	F0F1 ATP synthase subunit A [Mycobacterium avium subsp. paratuberculosis K-10]
gi|41408562	MAP2464c	Rho	transcription termination factor Rho	2		5.425620079	66871.55469	No signal peptide detected	transcription termination factor Rho [Mycobacterium avium subsp. paratuberculosis K-10]
gi|41408588	MAP2490	OppD		3		6.083420753	65347.76172	No signal peptide detected	OppD [Mycobacterium avium subsp. paratuberculosis K-10]
gi|41408591	MAP2493c			7		4.949806213	93140.36719	No signal peptide detected	hypothetical protein MAP2493c [Mycobacterium avium subsp. paratuberculosis K-10]
gi|41408634	MAP2536	kgd	alpha-ketoglutarate decarboxylase	7		5.58883667	137056.9375	No signal peptide detected	alpha-ketoglutarate decarboxylase [Mycobacterium avium subsp. paratuberculosis K-10]
gi|41408635	MAP2537		short-chain type dehydrogenase/reductase	7		9.393463135	28969.58594	No signal peptide detected	hypothetical protein MAP2537 [Mycobacterium avium subsp. paratuberculosis K-10]
gi|41408639	MAP2541c	mdh	malate dehydrogenase	7	1	4.592564583	34626.80078	No signal peptide detected	malate dehydrogenase [Mycobacterium avium subsp. paratuberculosis K-10]
gi|41408653	MAP2555c	HtrA		7	1	5.477033615	52147.72656	No signal peptide detected	HtrA [Mycobacterium avium subsp. paratuberculosis K-10]
gi|41408656	MAP2558		methyltransferase	7		4.229471207	24824.80664	No signal peptide detected	hypothetical protein MAP2558 [Mycobacterium avium subsp. paratuberculosis K-10]
gi|41408659	MAP2561		tetronasin-transport integral membrane protein ABC transporter	3	14	9.997221947	56363.05469	No signal peptide detected	hypothetical protein MAP2561 [Mycobacterium avium subsp. paratuberculosis K-10]
gi|41408660	MAP2562		integral membrane protein	3	2	9.932443619	18339.52734	No signal peptide detected	hypothetical protein MAP2562 [Mycobacterium avium subsp. paratuberculosis K-10]
gi|41408669	MAP2571c	FadD6	acyl-CoA synthetase	1		5.942954063	63787.29297	No signal peptide detected	acyl-CoA synthetase [Mycobacterium avium subsp. paratuberculosis K-10]
gi|41408696	MAP2598c		sulfate adenylyltransferase	11		5.844980717	47163.75781	No signal peptide detected	sulfate adenylyltransferase [Mycobacterium avium subsp. paratuberculosis K-10]
gi|41408735	MAP2637c		short-chain type dehydrogenase/reductase	7		5.667860031	23855.49414	No signal peptide detected	hypothetical protein MAP2637c [Mycobacterium avium subsp. paratuberculosis K-10]
gi|41408761	MAP2663c		no	11		9.716152191	10014.68262	No signal peptide detected	hypothetical protein MAP2663c [Mycobacterium avium subsp. paratuberculosis K-10]
gi|41408777	MAP2679c			10		10.34464455	16403.95898	No signal peptide detected	hypothetical protein MAP2679c [Mycobacterium avium subsp. paratuberculosis K-10]
gi|41408781	MAP2683			10	4	11.05390835	44302.98828	No signal peptide detected	hypothetical protein MAP2683 [Mycobacterium avium subsp. paratuberculosis K-10]
gi|41408783	MAP2685			10		4.590035439	21334.41406	No signal peptide detected	hypothetical protein MAP2685 [Mycobacterium avium subsp. paratuberculosis K-10]
gi|41408792	MAP2694			3	1	4.97946167	34795.60156	No signal peptide detected	hypothetical protein MAP2694 [Mycobacterium avium subsp. paratuberculosis K-10]
gi|41408796	MAP2698c	DesA2		1		4.627439499	31465.61133	No signal peptide detected	DesA2 [Mycobacterium avium subsp. paratuberculosis K-10]
gi|41408797	MAP2699c	glyA	serine hydroxymethyltransferase	7	1	5.691601753	44949.55078	No signal peptide detected	serine hydroxymethyltransferase [Mycobacterium avium subsp. paratuberculosis K-10]
gi|41408804	MAP2706c			10		4.958999157	71207.28906	No signal peptide detected	hypothetical protein MAP2706c [Mycobacterium avium subsp. paratuberculosis K-10]

gi|41408819	MAP2721			10		7.314506531	18019.26563	No signal peptide detected	hypothetical protein MAP2721 [Mycobacterium avium subsp. paratuberculosis K-10]
gi|41408935	MAP2837c			3	1	3.988495827	73800.26563	No signal peptide detected	hypothetical protein MAP2837c [Mycobacterium avium subsp. paratuberculosis K-10]
gi|41408950	MAP2852	ephG		0		5.147153854	15914.37207	No signal peptide detected	hypothetical protein MAP2852 [Mycobacterium avium subsp. paratuberculosis K-10]
gi|41408953	MAP2855c	35kd_ag		10		5.219333649	29484.41797	No signal peptide detected	35kd_ag [Mycobacterium avium subsp. paratuberculosis K-10]
gi|41408960	MAP2862			10		9.708192825	32939.11328	No signal peptide detected	hypothetical protein MAP2862 [Mycobacterium avium subsp. paratuberculosis K-10]
gi|41408989	MAP2891c	gpsI	polynucleotide phosphorylase/polyadenylase	2		4.456401348	80532.11719	No signal peptide detected	polynucleotide phosphorylase/polyadenylase [Mycobacterium avium subsp. paratuberculosis K-10]
gi|41409005	MAP2907c	infB	translation initiation factor IF-2	2		6.166002274	96513.41406	No signal peptide detected	translation initiation factor IF-2 [Mycobacterium avium subsp. paratuberculosis K-10]
gi|41409054	MAP2956c	rpsB	30S ribosomal protein S2	2		5.987771988	30149.63281	No signal peptide detected	30S ribosomal protein S2 [Mycobacterium avium subsp. paratuberculosis K-10]
gi|41409066	MAP2968		no	11		4.473490238	27227.39258	No signal peptide detected	hypothetical protein MAP2968 [Mycobacterium avium subsp. paratuberculosis K-10]
gi|41409075	MAP2977c		30S ribosomal protein S16	2		9.81638813	18103.35938	No signal peptide detected	30S ribosomal protein S16 [Mycobacterium avium subsp. paratuberculosis K-10]
gi|41409077	MAP2979	DacB		3	1	4.667075157	27117.04297	Signal peptide detected	DacB [Mycobacterium avium subsp. paratuberculosis K-10]
gi|41409088	MAP2990c	Smc	chromosome partition protein	3	1	5.007576942	133395.2656	No signal peptide detected	Smc [Mycobacterium avium subsp. paratuberculosis K-10]
gi|41409095	MAP2997c			10		4.535820007	26950.34766	No signal peptide detected	hypothetical protein MAP2997c [Mycobacterium avium subsp. paratuberculosis K-10]
gi|41409105	MAP3007		oxidoreductase	7		4.269639969	30009.58594	No signal peptide detected	hypothetical protein MAP3007 [Mycobacterium avium subsp. paratuberculosis K-10]
gi|41409122	MAP3024c	HupB		2				No signal peptide detected	HupB [Mycobacterium avium subsp. paratuberculosis K-10]
gi|41409131	MAP3033c	SerA		7		4.599835873	54501.08594	No signal peptide detected	SerA [Mycobacterium avium subsp. paratuberculosis K-10]
gi|41409138	MAP3040c			10		6.478748322	22889.5625	No signal peptide detected	hypothetical protein MAP3040c [Mycobacterium avium subsp. paratuberculosis K-10]
gi|41409158	MAP3060c	FixB		7		4.3382864	27812.73633	Signal peptide detected	FixB [Mycobacterium avium subsp. paratuberculosis K-10]
gi|41409188	MAP3090c	SerB2		7		4.543779373	43432.4375	No signal peptide detected	SerB2 [Mycobacterium avium subsp. paratuberculosis K-10]
gi|41409196	MAP3098c		monooxygenase	7	1	6.848970413	57025.73828	No signal peptide detected	hypothetical protein MAP3098c [Mycobacterium avium subsp. paratuberculosis K-10]
gi|41409198	MAP3100c	nrdE	ribonucleotide-diphosphate reductase subunit alpha	2		5.635979176	82366.02344	No signal peptide detected	ribonucleotide-diphosphate reductase subunit alpha [Mycobacterium avium subsp. paratuberculosis K-10]
gi|41409269	MAP3171c	FtsX		3	1	7.140640259	27917.97266	No signal peptide detected	FtsX [Mycobacterium avium subsp. paratuberculosis K-10]
gi|41409270	MAP3172c	FtsE		3		9.941630363	25682.53711	No signal peptide detected	FtsE [Mycobacterium avium subsp. paratuberculosis K-10]
gi|41409286	MAP3188	FadE24		1	1	5.600812912	49533.15234	No signal peptide detected	FadE24 [Mycobacterium avium subsp. paratuberculosis K-10]
gi|41409287	MAP3189	FadE23		1		4.974378586	43321.73438	No signal peptide detected	FadE23 [Mycobacterium avium subsp. paratuberculosis K-10]
gi|41409297	MAP3199		no	11		7.182891846	19694.12695	No signal peptide detected	hypothetical protein MAP3199 [Mycobacterium avium subsp. paratuberculosis K-10]
gi|41409298	MAP3200		response regulator	9		5.394042969	14787.24023	No signal peptide detected	hypothetical protein MAP3200 [Mycobacterium avium subsp. paratuberculosis K-10]
gi|41409302	MAP3204	nuoD	NADH dehydrogenase	7	3	4.929944992	48352.49219	No signal peptide detected	NADH dehydrogenase subunit D [Mycobacterium avium subsp. paratuberculosis K-10]

			subunit D						
gi|41409305	MAP3207		NADH dehydrogenase subunit G	7	1	4.814846992	82378.92969	No signal peptide detected	NADH dehydrogenase subunit G [Mycobacterium avium subsp. paratuberculosis K-10]
gi|41409307	MAP3209		NADH dehydrogenase subunit I	7		5.440038681	20216.45898	Signal peptide detected	NADH dehydrogenase subunit I [Mycobacterium avium subsp. paratuberculosis K-10]
gi|41409310	MAP3212		NADH dehydrogenase subunit L	7	14	8.149744034	66282.96875	No signal peptide detected	NADH dehydrogenase subunit L [Mycobacterium avium subsp. paratuberculosis K-10]
gi|41409331	MAP3233c			10		4.539906979	69172.29688	No signal peptide detected	hypothetical protein MAP3233c [Mycobacterium avium subsp. paratuberculosis K-10]
gi|41409358	MAP3260			10	1	9.332094193	34968.76563	No signal peptide detected	hypothetical protein MAP3260 [Mycobacterium avium subsp. paratuberculosis K-10]
gi|41409370	MAP3272			10		7.205307007	15545.91699	No signal peptide detected	hypothetical protein MAP3272 [Mycobacterium avium subsp. paratuberculosis K-10]
gi|41409380	MAP3282c	ephD	short chain dehydrogenase	0		8.485054016	33090.21484	No signal peptide detected	hypothetical protein MAP3282c [Mycobacterium avium subsp. paratuberculosis K-10]
gi|41409382	MAP3284	FadD29		1	2	5.340837955	123196.25	No signal peptide detected	FadD29 [Mycobacterium avium subsp. paratuberculosis K-10]
gi|41409383	MAP3285c	dxr	1-deoxy-D-xylulose 5-phosphate reductoisomerase	7		10.31865883	37975.01953	No signal peptide detected	hypothetical protein MAP3285c [Mycobacterium avium subsp. paratuberculosis K-10]
gi|41409385	MAP3287		short chain dehydrogenase	7		9.792224884	31624.03711	No signal peptide detected	hypothetical protein MAP3287 [Mycobacterium avium subsp. paratuberculosis K-10]
gi|41409389	MAP3291c			3	1	8.702994347	103680.5391	No signal peptide detected	hypothetical protein MAP3291c [Mycobacterium avium subsp. paratuberculosis K-10]
gi|41409403	MAP3305c			10	1	6.69952774	30748.49805	No signal peptide detected	hypothetical protein MAP3305c [Mycobacterium avium subsp. paratuberculosis K-10]
gi|41409412	MAP3314c		SOJ/PARA-like protein	3		6.226428986	28101.0957	No signal peptide detected	hypothetical protein MAP3314c [Mycobacterium avium subsp. paratuberculosis K-10]
gi|41409423	MAP3325		short chain dehydrogenase	7		4.882461548	29808.58203	No signal peptide detected	short chain dehydrogenase [Mycobacterium avium subsp. paratuberculosis K-10]
gi|41409452	MAP3354c	secA	preprotein translocase subunit SecA	3		4.997069359	105230.9766	No signal peptide detected	preprotein translocase subunit SecA [Mycobacterium avium subsp. paratuberculosis K-10]
gi|41409458	MAP3360c	MtrA		9		5.106352806	25262.23242	No signal peptide detected	MtrA [Mycobacterium avium subsp. paratuberculosis K-10]
gi|41409460	MAP3362c	sahH	S-adenosyl-L-homocysteine hydrolase	7		4.721640587	54428.58984	No signal peptide detected	S-adenosyl-L-homocysteine hydrolase [Mycobacterium avium subsp. paratuberculosis K-10]
gi|41409467	MAP3369c	manB	phosphomannomutase/phosphoglucomutase	3	1	4.378991127	49260.74219	No signal peptide detected	phosphomannomutase/phosphoglucomutase [Mycobacterium avium subsp. paratuberculosis K-10]
gi|41409526	MAP3428c	cut3	cutinase precursor CUT3	3	1	4.479298592	19234.07227	No signal peptide detected	hypothetical protein MAP3428c [Mycobacterium avium subsp. paratuberculosis K-10]
gi|41409540	MAP3442	SdhD		7	2	9.734773636	18000.88867	No signal peptide detected	SdhD [Mycobacterium avium subsp. paratuberculosis K-10]
gi|41409554	MAP3456c	Icd2		7		5.815195084	83070.60156	No signal peptide detected	Icd2 [Mycobacterium avium subsp. paratuberculosis K-10]
gi|41409563	MAP3465			3	6	9.38743782	93290.32031	No signal peptide detected	hypothetical protein MAP3465 [Mycobacterium avium subsp. paratuberculosis K-10]
gi|41409570	MAP3472c			10	3	6.413895607	99879.0625	No signal peptide detected	hypothetical protein MAP3472c [Mycobacterium avium subsp. paratuberculosis K-10]
gi|41409596	MAP3498c	CtpI		3		5.112781048	161918.9688	No signal peptide detected	CtpI [Mycobacterium avium subsp. paratuberculosis K-10]
gi|41409613	MAP3515c		low	7		4.399828911	22180.28711	No signal peptide detected	hypothetical protein MAP3515c [Mycobacterium avium subsp. paratuberculosis K-10]

gi|41409625	MAP3527	PepA		7	1	4.275987625	31712.19141	Signal peptide detected	PepA [Mycobacterium avium subsp. paratuberculosis K-10]
gi|41409629	MAP3531c	FbpC2		1	1	4.717447281	33402.15625	Signal peptide detected	FbpC2 [Mycobacterium avium subsp. paratuberculosis K-10]
gi|41409643	MAP3545		low	7		6.320118904	38842.00391	No signal peptide detected	hypothetical protein MAP3545 [Mycobacterium avium subsp. paratuberculosis K-10]
gi|41409644	MAP3546	DesA3_2		1	3	6.575153351	41964.20313	No signal peptide detected	DesA3_2 [Mycobacterium avium subsp. paratuberculosis K-10]
gi|41409664	MAP3566		aldehyde dehydrogenase	7	1	9.390995979	53030.44531	No signal peptide detected	hypothetical protein MAP3566 [Mycobacterium avium subsp. paratuberculosis K-10]
gi|41409665	MAP3567		short-chain type dehydrogenase/reductase	7		5.620178223	30180.38477	No signal peptide detected	hypothetical protein MAP3567 [Mycobacterium avium subsp. paratuberculosis K-10]
gi|41409675	MAP3577	FabG3_2		1		5.348567963	25918.44141	No signal peptide detected	FabG3_2 [Mycobacterium avium subsp. paratuberculosis K-10]
gi|41409696	MAP3598			10		4.312573433	17027.32617	No signal peptide detected	hypothetical protein MAP3598 [Mycobacterium avium subsp. paratuberculosis K-10]
gi|41409707	MAP3609	mce1F	MCE-family protein MCE1F	10		5.066803932	54547.91016	No signal peptide detected	hypothetical protein MAP3609 [Mycobacterium avium subsp. paratuberculosis K-10]
gi|41409710	MAP3612		MCE associated protein	10	1	4.534803391	14148.85449	No signal peptide detected	hypothetical protein MAP3612 [Mycobacterium avium subsp. paratuberculosis K-10]
gi|41409732	MAP3634			10				Signal peptide detected	hypothetical protein MAP3634 [Mycobacterium avium subsp. paratuberculosis K-10]
gi|41409739	MAP3641c	MmpL3		3	12	9.640067101	102309.1953	Signal peptide detected	MmpL3 [Mycobacterium avium subsp. paratuberculosis K-10]
gi|41409749	MAP3651c	FadE3_2		1	1	6.1208992	44045.71484	No signal peptide detected	FadE3_2 [Mycobacterium avium subsp. paratuberculosis K-10]
gi|41409757	MAP3659		low	1	1	4.311993599	53743.91797	No signal peptide detected	hypothetical protein MAP3659 [Mycobacterium avium subsp. paratuberculosis K-10]
gi|41409764	MAP3666c			3	2	4.529676914	44720.12109	No signal peptide detected	hypothetical protein MAP3666c [Mycobacterium avium subsp. paratuberculosis K-10]
gi|41409789	MAP3691c			7	1	9.92843914	30368.3125	No signal peptide detected	hypothetical protein MAP3691c [Mycobacterium avium subsp. paratuberculosis K-10]
gi|41409790	MAP3692c	fabG	3-ketoacyl-(acyl-carrier-pr otein) reductase	1		5.696573257	47261.84766	No signal peptide detected	3-ketoacyl-(acyl-carrier-protein) reductase [Mycobacterium avium subsp. paratuberculosis K-10]
gi|41409791	MAP3693	fadA2	acetyl-CoA acetyltransferase	1		6.195707321	46844.14063	No signal peptide detected	acetyl-CoA acetyltransferase [Mycobacterium avium subsp. paratuberculosis K-10]
gi|41409795	MAP3697c		fumarate reductase iron-sulfur subunit	7		5.973936081	28635.30469	No signal peptide detected	fumarate reductase iron-sulfur subunit [Mycobacterium avium subsp. paratuberculosis K-10]
gi|41409796	MAP3698c	sdhA	succinate dehydrogenase flavoprotein subunit	7		5.608189583	70715.25	No signal peptide detected	succinate dehydrogenase flavoprotein subunit [Mycobacterium avium subsp. paratuberculosis K-10]
gi|41409797	MAP3699c		succinate dehydrogenase membrane anchor subunit	7	5	9.610275269	31616.06055	No signal peptide detected	hypothetical protein MAP3699c [Mycobacterium avium subsp. paratuberculosis K-10]
gi|41409929	MAP3831c		iron-sulfur-binding reductase	7	5	6.55079174	103037.7969	No signal peptide detected	hypothetical protein MAP3831c [Mycobacterium avium subsp. paratuberculosis K-10]
gi|41409938	MAP3840	DnaK	molecular chaperone DnaK	0		4.444113731	66509.80469	No signal peptide detected	molecular chaperone DnaK [Mycobacterium avium subsp. paratuberculosis K-10]
gi|41409939	MAP3841	GrpE		0		4.284734249	23705.70117	No signal peptide detected	GrpE [Mycobacterium avium subsp. paratuberculosis K-10]
gi|41409940	MAP3842	DnaJ		0		8.318973541	41274.82813	No signal peptide detected	DnaJ [Mycobacterium avium subsp. paratuberculosis K-10]
gi|41409941	MAP3843	HspR		0		10.60127831	14654.97363	No signal peptide detected	HspR [Mycobacterium avium subsp. paratuberculosis K-10]
gi|41409942	MAP3844		no	11	1	5.936054707	154779.7969	No signal peptide detected	hypothetical protein MAP3844 [Mycobacterium avium subsp. paratuberculosis K-10]

gi|41409951	MAP3853	ClpB		0		4.963713646	89422.83594	No signal peptide detected	ClpB [Mycobacterium avium subsp. paratuberculosis K-10]
gi|41409975	MAP3877c	FadE20_3		1		5.180764198	42007.44141	No signal peptide detected	FadE20_3 [Mycobacterium avium subsp. paratuberculosis K-10]
gi|41409983	MAP3885		phosphate acetyltransferase	7		5.197104454	73529.88281	No signal peptide detected	phosphate acetyltransferase [Mycobacterium avium subsp. paratuberculosis K-10]
gi|41410026	MAP3928c		putative ATPase	3	1	4.789388657	74663.75781	No signal peptide detected	hypothetical protein MAP3928c [Mycobacterium avium subsp. paratuberculosis K-10]
gi|41410028	MAP3930c	psd	phosphatidylserine decarboxylase	1		10.41528702	25047.91406	No signal peptide detected	phosphatidylserine decarboxylase [Mycobacterium avium subsp. paratuberculosis K-10]
gi|41410031	MAP3933c		short chain dehydrogenase	7		6.77684021	33774.11328	No signal peptide detected	short chain dehydrogenase [Mycobacterium avium subsp. paratuberculosis K-10]
gi|41410034	MAP3936		chaperonin GroEL	0		4.60484457	56635.20703	No signal peptide detected	chaperonin GroEL [Mycobacterium avium subsp. paratuberculosis K-10]
gi|41410066	MAP3968	hbhA	iron-regulated heparin binding hemagglutinin hbhA (adhesin)	3		8.524131775	21288.375	No signal peptide detected	hypothetical protein MAP3968 [Mycobacterium avium subsp. paratuberculosis K-10]
gi|41410089	MAP3991	proC	pyrroline-5-carboxylate reductase	7		4.562462807	30388.87109	No signal peptide detected	pyrroline-5-carboxylate reductase [Mycobacterium avium subsp. paratuberculosis K-10]
gi|41410093	MAP3995c	CmaA2		1		5.018967152	34221.76953	No signal peptide detected	CmaA2 [Mycobacterium avium subsp. paratuberculosis K-10]
gi|41410095	MAP3997c	SerB		7	1	5.956876755	32257.39453	No signal peptide detected	SerB [Mycobacterium avium subsp. paratuberculosis K-10]
gi|41410116	MAP4018c			10		11.50753021	15409.00879	No signal peptide detected	hypothetical protein MAP4018c [Mycobacterium avium subsp. paratuberculosis K-10]
gi|41410124	MAP4026			10		5.859455109	46325.48438	No signal peptide detected	hypothetical protein MAP4026 [Mycobacterium avium subsp. paratuberculosis K-10]
gi|41410156	MAP4058	GrcC1		7		4.800927639	36134.39453	No signal peptide detected	GrcC1 [Mycobacterium avium subsp. paratuberculosis K-10]
gi|41410201	MAP4103c			3		5.42195034	27143.00977	No signal peptide detected	hypothetical protein MAP4103c [Mycobacterium avium subsp. paratuberculosis K-10]
gi|41410208	MAP4110	secE	preprotein translocase subunit SecE	3	1	9.946502686	15874.14453	No signal peptide detected	preprotein translocase subunit SecE [Mycobacterium avium subsp. paratuberculosis K-10]
gi|41410211	MAP4113		50S ribosomal protein L1	2		10.09300709	24895.39453	No signal peptide detected	50S ribosomal protein L1 [Mycobacterium avium subsp. paratuberculosis K-10]
gi|41410224	MAP4126		50S ribosomal protein L7/L12	2		4.675282478	7700.880371	No signal peptide detected	50S ribosomal protein L7/L12 [Mycobacterium avium subsp. paratuberculosis K-10]
gi|41410227	MAP4129	mkl	ribonucleotide ABC transporter ATP-binding protein	3		5.071614265	36669.72266	No signal peptide detected	hypothetical protein MAP4129 [Mycobacterium avium subsp. paratuberculosis K-10]
gi|41410228	MAP4130		DNA-directed RNA polymerase subunit beta	2		4.686635971	132032.1875	No signal peptide detected	DNA-directed RNA polymerase subunit beta [Mycobacterium avium subsp. paratuberculosis K-10]
gi|41410229	MAP4131		DNA-directed RNA polymerase subunit beta'	2		5.775175095	146997.9844	No signal peptide detected	DNA-directed RNA polymerase subunit beta' [Mycobacterium avium subsp. paratuberculosis K-10]
gi|41410239	MAP4141		rpsG 30S ribosomal protein S7	2		11.11026859	17598.30859	No signal peptide detected	30S ribosomal protein S7 [Mycobacterium avium subsp. paratuberculosis K-10]
gi|41410241	MAP4143	Tuf	elongation factor Tu	2		4.964131832	43765.28125	No signal peptide detected	elongation factor Tu [Mycobacterium avium subsp. paratuberculosis K-10]
gi|41410243	MAP4145			3	2	7.196182251	30045.08594	No signal peptide detected	hypothetical protein MAP4145 [Mycobacterium avium subsp. paratuberculosis K-10]
gi|41410252	MAP4154	LldD1		7		6.640068054	41849.93359	No signal peptide detected	LldD1 [Mycobacterium avium subsp. paratuberculosis K-10]

gi|41410259	MAP4161		50S ribosomal protein L3	2		10.79018974	23080.62695	No signal peptide detected	50S ribosomal protein L3 [Mycobacterium avium subsp. paratuberculosis K-10]
gi|41410262	MAP4164		50S ribosomal protein L2	2		11.72452831	30389.94727	No signal peptide detected	50S ribosomal protein L2 [Mycobacterium avium subsp. paratuberculosis K-10]
gi|41410265	MAP4167		30S ribosomal protein S3	2		10.58676052	30583.79102	No signal peptide detected	30S ribosomal protein S3 [Mycobacterium avium subsp. paratuberculosis K-10]
gi|41410266	MAP4168		50S ribosomal protein L16	2		11.6141634	15719.44629	No signal peptide detected	50S ribosomal protein L16 [Mycobacterium avium subsp. paratuberculosis K-10]
gi|41410268	MAP4170		30S ribosomal protein S17	2		10.8671236	13025.78418	No signal peptide detected	30S ribosomal protein S17 [Mycobacterium avium subsp. paratuberculosis K-10]
gi|41410277	MAP4179		50S ribosomal protein L5	2		10.34163189	21092.6582	No signal peptide detected	50S ribosomal protein L5 [Mycobacterium avium subsp. paratuberculosis K-10]
gi|41410279	MAP4181		30S ribosomal protein S8	2	1	10.47575092	14423.85449	No signal peptide detected	30S ribosomal protein S8 [Mycobacterium avium subsp. paratuberculosis K-10]
gi|41410280	MAP4182		50S ribosomal protein L6	2		10.63413239	19451.96289	No signal peptide detected	50S ribosomal protein L6 [Mycobacterium avium subsp. paratuberculosis K-10]
gi|41410328	MAP4230		30S ribosomal protein S13	2		11.3183527	14306.88574	No signal peptide detected	30S ribosomal protein S13 [Mycobacterium avium subsp. paratuberculosis K-10]
gi|41410330	MAP4232		30S ribosomal protein S4	2		10.35540581	23492.11719	No signal peptide detected	30S ribosomal protein S4 [Mycobacterium avium subsp. paratuberculosis K-10]
gi|41410331	MAP4233		DNA-directed RNA polymerase subunit alpha	2		4.404465675	37699.63672	No signal peptide detected	DNA-directed RNA polymerase subunit alpha [Mycobacterium avium subsp. paratuberculosis K-10]
gi|41410343	MAP4245		50S ribosomal protein L13	2		10.37167168	16164.77734	No signal peptide detected	50S ribosomal protein L13 [Mycobacterium avium subsp. paratuberculosis K-10]
gi|41410348	MAP4250c		coenzyme F420-dependent oxidoreductase	7		6.507263184	37766.97656	No signal peptide detected	hypothetical protein MAP4250c [Mycobacterium avium subsp. paratuberculosis K-10]
gi|41410349	MAP4251c			10		10.26061821	28791.85547	Signal peptide detected	hypothetical protein MAP4251c [Mycobacterium avium subsp. paratuberculosis K-10]
gi|41410363	MAP4265	GroEL		0		4.608287811	55786.01953	No signal peptide detected	chaperonin GroEL [Mycobacterium avium subsp. paratuberculosis K-10]
gi|41410434	MAP4336		transmembrane protein	3	1	9.702390671	116695.8672	No signal peptide detected	hypothetical protein MAP4336 [Mycobacterium avium subsp. paratuberculosis K-10]
